# Fast rate of evolution in alternatively spliced coding regions of mammalian genes

**DOI:** 10.1186/1471-2164-7-84

**Published:** 2006-04-18

**Authors:** Ekaterina O Ermakova, Ramil N Nurtdinov, Mikhail S Gelfand

**Affiliations:** 1 Department of Bioengineering and Bioinformatics, Moscow State University, Vorob'evy gory, 1-73, 119992, Moscow, Russia; 2 Research and Training Center "Bioinformatics", Institute for Information Transmission Problems, Russian Academy of Sciences, Bolshoi Karetny per. 19, 127994, Moscow, Russia

## Abstract

**Background:**

At least half of mammalian genes are alternatively spliced. Alternative isoforms are often genome-specific and it has been suggested that alternative splicing is one of the major mechanisms for generating protein diversity in the course of evolution. Another way of looking at alternative splicing is to consider sequence evolution of constitutive and alternative regions of protein-coding genes. Indeed, it turns out that constitutive and alternative regions evolve in different ways.

**Results:**

A set of 3029 orthologous pairs of human and mouse alternatively spliced genes was considered. The rate of nonsynonymous substitutions (d_N_), the rate of synonymous substitutions (d_S_), and their ratio (ω = d_N_/d_S_) appear to be significantly higher in alternatively spliced coding regions compared to constitutive regions. When N-terminal, internal and C-terminal alternatives are analysed separately, C-terminal alternatives appear to make the main contribution to the observed difference. The effects become even more pronounced in a subset of fast evolving genes.

**Conclusion:**

These results provide evidence of weaker purifying selection and/or stronger positive selection in alternative regions and thus one more confirmation of accelerated evolution in alternative regions. This study corroborates the theory that alternative splicing serves as a testing ground for molecular evolution.

## Background

Alternative splicing is a major mechanism for generating functional and evolutionary diversity of proteins in mammals [1,2], for a review see [3]. Indeed, alternative splicing allows for generation of novel proteins without sacrificing old ones [2]. If a new isoform proves to be beneficial, its fraction increases by subtle regulatory changes. On the other hand, unlike gene duplication, alternative splicing does not lead to dramatic changes in protein concentrations. Moreover, it has been demonstrated that duplicated genes are rarely alternatively spliced compared to singletons [4,5].

There are good reasons to believe that some key mutational events driving evolution might reside in introns, untranslated regions (UTRs) and/or nontranscribed regulatory regions [6-8]. A large fraction of alternative splicing events occur in untranslated regions [9]. Nevertheless, most studies of molecular evolution have focused on the analysis of protein coding regions, as these data are simpler to obtain and are more amenable to functional interpretation.

From this point of view, alternative regions of genes occupy an intermediate position. Alternatively spliced regions are often evolutionary young: indeed, about a half of genes in human-mouse orthologous pairs have species-specific isoforms [2,10]. In many respects, constitutive and alternative regions are organized in different ways. Alternative human splice sites are on the average weaker than constitutive ones [11]. Non-canonical GC-AG introns tend to be alternative [12]. Among human exons conserved in mouse, about 77% of alternative cassette exons are flanked on both sides by long conserved intronic sequences, compared to only 17% of the constitutive exons [13]. Overall, statistical and evolutionary features of constitutive and alternative exons are sufficiently different to provide for computational recognition of these exons [14-16].

In several early studies it has been observed that patterns of nucleotide substitutions are different in alternative and constitutive coding regions. Iida and Akashi [17] analysed 26 pairs of alternatively spliced human genes and their non-human mammalian orthologs and demonstrated that synonymous divergence was lower and the nonsynonymous divergence was higher in alternative regions compared to constitutive regions. Evidence for diversifying selection was observed in alternative regions of CD45 [18], whereas the reduced rate of synonymous substitutions in an alternative region of BRCA1 [19] was assigned to purifying selection due to exonic splicing enhancer sites [20]. Recently, lower synonymous divergence in alternative exons compared to constitutive ones was demonstrated in a large-scale study of human, chimpanzee, mouse and rat genes [21].

Here we analyze evolutionary patterns in a set of 3029 pairs of orthologous human and mouse genes. We consider all types of alternative splicing and analyze separately 5'-, internal, and 3'-regions of genes, as well as faster and slower evolving genes.

## Results

**Figure 1:**
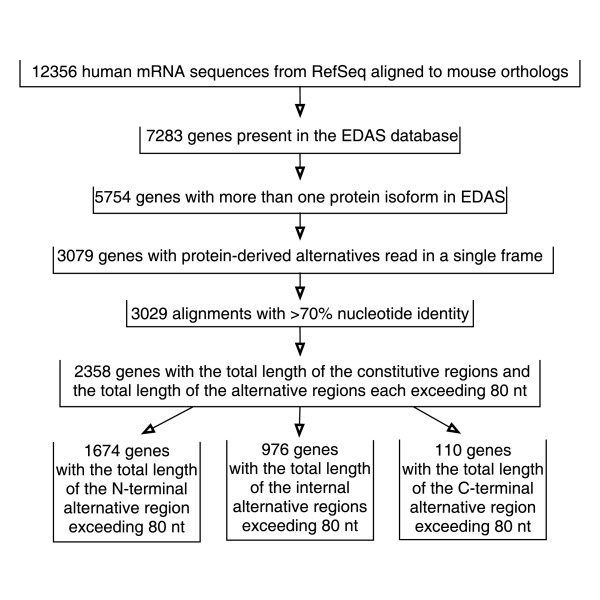
The data flow through the analysis pipeline.

**Figure 2:**
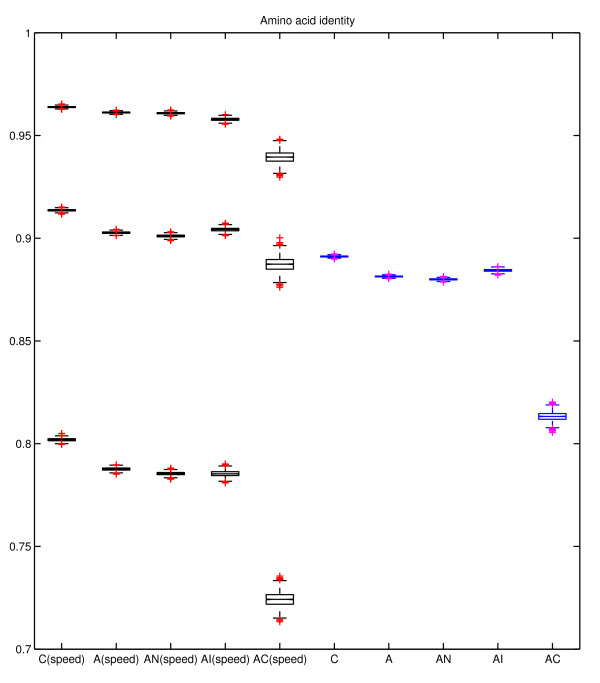
**Amino acid identity in alignments of constitutive and alternative coding regions of 3029 alternatively spliced genes classified by the overall evolution rate**. C – constitutive regions. A – alternative regions. AN – N-terminal alternative regions. AI – internal alternative regions. AC – C-terminal alternative regions. Five columns on the left show results for three equal bins of slowly evolving genes, genes evolving with medium speed, and fast evolving genes. Five columns on the right correspond to the total sample of 3029 alternatively spliced genes. To estimate the average amino acid identity, we used global meta-alignments (see Methods). The boxplots represent the results for 2000 bootstrap replications.

**Figure 3:**
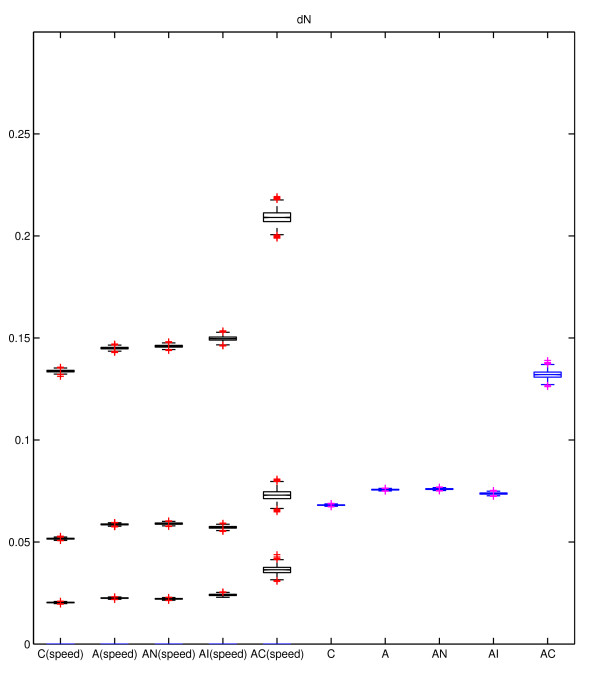
**Nonsynonymous substitution rate (d_N_) in alignments of constitutive and alternative coding regions of 3029 alternatively spliced genes genes classified by the overall evolution rate**. Notation and layout as in Figure 2.

**Figure 4:**
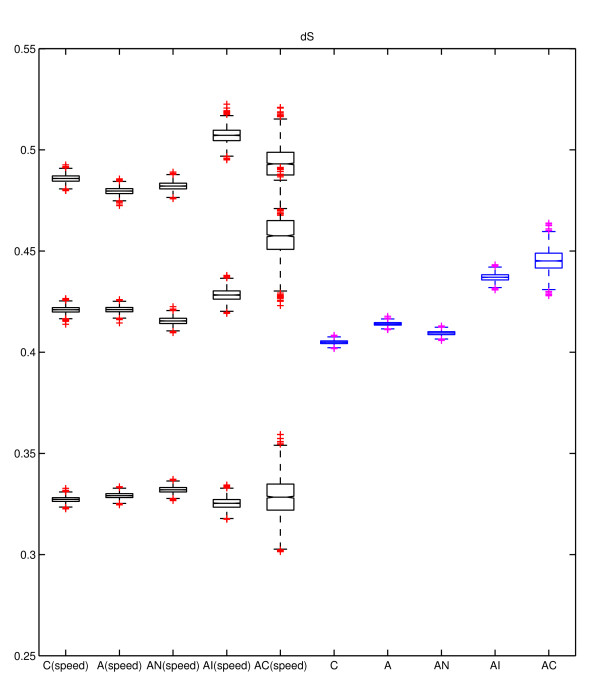
**Synonymous substitution rate (d_S_) in alignments of constitutive and alternative coding regions of 3029 alternatively spliced genes classified by the overall evolution rate**. Notation and layout as in Figure 2.

**Figure 5:**
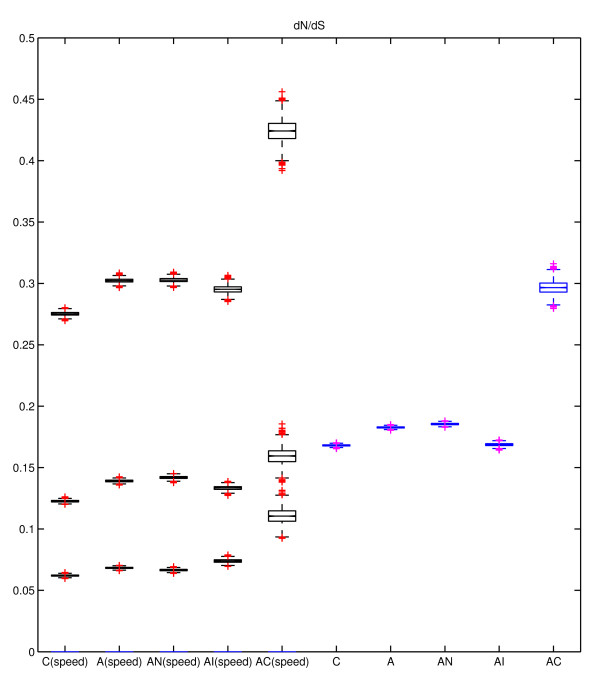
**Selection measure ω = d_N_/d_S _in alignments of constitutive and alternative coding regions of 3029 alternatively spliced genes classified by the overall evolution rate**. Notation and layout as in Figure 2.

We considered 3029 alternatively spliced human genes and their mouse orthlogs (Figure 1). The sample was divided into three bins of equal size with respect to nucleotide identity in coding regions. Nucleotide alignments of coding regions were sliced into constitutive (C) and alternative (A) fragments. Alternative fragments were further sorted into N-terminal (AN), internal (AI), and C-terminal (AC). To reveal the general pattern of evolution in these regions, we estimated the amino acid identity (Id), the nonsynonymous substitution rate (d_N_), the synonymous substitution rate (d_S_), and ω = d_N_/d_S _(Table 1). Global meta-alignments (concatenated alignment fragments across all considered genes and across three rate bins, see Methods) of five types (C, A, AN, AI, AC) were used. We performed 2000 bootstrap replications to evaluate the robustness of our estimates (Figures 2, 3, 4, 5).

It turned out that Id(A)<Id(C), d_N_(A)>d_N_(C), and ω(A)>ω(C) for alternatively spliced genes irrespective of the rate of evolution. These results show that negative selection is weaker and/or positive selection is stronger in alternative regions and thus confirm that alternatively spliced coding regions are hotspots of molecular evolution. Unexpectedly, d_N _and ω rise dramatically at the C-terminal alternative regions (Figures 3 and 5).

The pattern of synonymous substitutions is more complicated, as it depends on the rate of evolution (Figure 4). The general pattern is that d_S _in alternative regions increases in the 5' to 3' direction. In genes evolving at the medium rate, d_S_(AN)<d_S_(AI)<d_S_(AC), whereas in fast evolving genes d_S_(AI)>d_S_(AC).

For control, we considered N-terminal and C-terminal constitutively spliced regions and performed similar analysis. All computed evolutionary parameters were the same as in the constitutive regions in general (data not shown). Thus the observed difference cannot be explained simply by faster evolution at gene termini.

**Figure 6:**
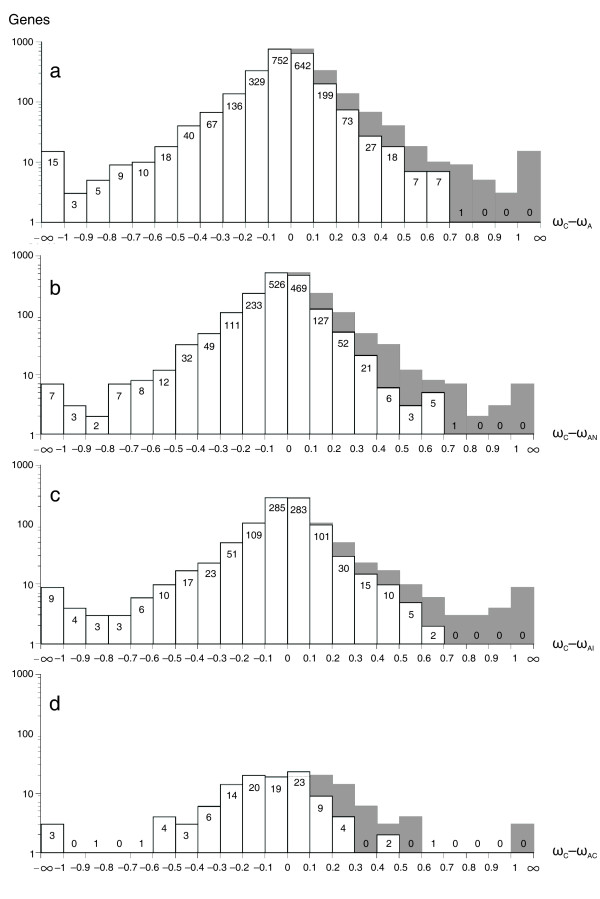
**Distributions of ω_C_-ω_A_, ω_C_-ω_AN_, ω_C_-ω_AI_, ω_C_-ω_AC_**. **a**. The distribution of ω_C_-ω_A _for 2358 genes with the total length of human-mouse alignments of constitutive regions and of alternative regions both exceeding 80 bp shows that ω tends to be larger in alternative regions. In particular, there are 23 genes with ω_C_-ω_A_<-0.8 and no genes with ω_C_-ω_A_>0.8. **b, c, d**. The distributions of ω_C_-ω_AN_, ω_C_-ω_AI_, ω_C_-ω_AC _for genes with long N-terminal (1674 genes, top, ω_C_-ω_AN_), internal (976 genes, middle, ω_C_-ω_AI_), and C-terminal (110 genes, bottom, ω_C_-ω_AC_) alternative regions, respectively, show that ω_A* _tends to be larger than ω_c _in all types of alternative regions. the grey shadows are symmetrical to the left parts of the histograms.

Next, we analyzed individual gene pairs. For each of 2358 genes with the total lengths of constitutive and of alternative regions both exceeding 80 bp, ω for constitutive (ω_C_) and alternative (ω_A_) regions was calculated separately. Figure 6a represents the distribution of the difference (ω_C_-ω_A_). The distribution is skewed, showing that ω tends to be greater in alternative regions. We used the chi-squared test to compare the distributions of |ω_C_-ω_A_| in the case ω_C_>ω_A _and in the case ω_C_<ω_A_. The null hypothesis that the distributions were the same was rejected at the significance level 10^-7^. When N-terminal, internal, and C-terminal alternatives were considered separately, the effect was the same (Figure 6b–d). The null hypothesis that the distribution of |ω_C_-ω_AN_| was symmetrical was rejected at the significance level 10^-9^, the one that the distribution of |ω_C_-ω_AI_| was symmetrical, at the significance level 10^-3^, and the one that the distribution of |ω_C_-ω_AC_| was symmetrical, at the significance level 10^-2^. Therefore, the detailed analysis of individual genes confirmed the observations made on concatenated alignments.

## Discussion

Evolutionary patterns in different functional regions are known to be significantly different. Conserved genes are duplicated relatively more often [22], although shortly after duplication the evolutionary rate might increase, as the purifying selection is weaker [23,24], and the selection pattern in the two copies may be different [25]. Duret and Mouchiroud [26] observed lower nonsynonymous divergence in genes expressed in multiple tissues when compared to genes with more limited expression patterns, whereas the synonymous substitution rate was roughly the same. Similarly, Pál, Papp and Hurst [27] demonstrated that highly expressed genes tend to be more conserved then genes expressed at a lower level. Our results are consistent with these observations if one assumes that constitutive regions are expressed in more tissues and at a higher rate that alternative ones. Indeed, the former assumption holds for genes with isoforms having clear tissue specific expression pattern, whereas the latter holds for genes with all isoforms expressed ubiquitously.

Young gene regions tend to evolve faster. Several studies [23-25,28] demonstrated post-duplicational relaxation of purifying selection in paralogs. Our results provided evidence of stronger positive selection and/or weaker purifying selection in alternative gene regions.

One possible explanation for our observations could be that the data are contaminated by non-functional isoforms (hence relaxation of purifying selection takes place). We do not believe that to be the case for the following reasons. Firstly, these regions were conserved between human and mouse at a sufficiently high similarity level of 70% nucleotide identity. Secondly, the observed pattern of increased d_N _level in alternative regions was the most pronounced in 3' (C-terminal) regions, that are the most reliable as regards gene recognition and have higher EST coverage due to polyA-primed ESTs.

As we considered only alternatives derived from RefSeq proteins, we could miss some alternatives and thus label a fraction of the alternative regions as constitutive. However, that could only contaminate the constitutive sample with alternative regions and thus blur the observed differences, but not create any spurious effect.

Recently Xing and Lee [21] observed similar rate of non-synonymous substitutions in alternative and constitutive regions whereas the rate of synonymous substitutions was lower in alternative regions, especially in tissue-specific exons [29]. One possible explanation for that was based on the assumption that conserved alternative exons contain more candidate splicing enhancer sites than constitutive ones [15]. As such sites could be expected to be conserved, like in BRCA1 [19,20], that could lead to higher conservation of synonymous codon positions in alternative regions compared to constitutive ones. However, this explanation seems to be incorrect, since, although indeed d_S _is lower in splicing enhancers, the fractions of constitutive and alternative regions covered by splicing enhancers are the same [30], and if the RNA selection pressure is the same in alternative and constitutive regions, it cannot distort the measurement of ω [31].

However, this effect has not been observed in our study, and the substitution rates differ from those in [21]. As our results are consistent and statistically significant for all classes of genes (fast, medium and slow evolving) and all gene regions (N-terminal, internal, C-terminal), and do not seem to be caused by contamination, there should be other reasons for this discrepancy. One of them could be the fact that we considered all types of alternatives, as opposed to only cassette exons in other studies. We also considered short alternative regions, its skipping or inclusion might be regulated "outside". Another one could be the use of different methods to calculate the rates of evolution. We used our own implementation of the first method of Ina [32] here, as we needed a tool for very long alignments (~ 3·10^6 ^bp), whereas Xing and Lee [21,29] used a maximum likelihood method implemented in the PAML package [33]. On the other hand, we considered only RefSeq isoforms and did not distinguish between the minor and major isoform alternatives.

An explanation for our finding could be that the total length of regulatory sites experiencing purifying selection is still small compared to the total length of alternative regions. The pattern of substitutions in insects is less consistent [34]: in N-terminal alternatives, the synonymous rate is higher than in constant regions, whereas in internal alternatives, there are more amino acid substitutions, similar to our observations here.

## Conclusion

Overall, this study corroborates the idea that alternative splicing serves as a testing ground for molecular evolution. Several lines of evidence confirm this hypothesis: (i) alternatively spliced isoforms are often evolutionary young both in mammals [2,10] and in insects [35]; (ii) the rate of nonsynonymous substitutions is higher in alternative regions compared to constitutive ones (this study), (iii) constitutive exons in genes with genome-specific alternative splicing evolve faster than constitutive regions in genes with conserved structure [36] (cf. a similar observation for duplicated genes [23-25]), (iv) many young (rodent-specific, missing in human and pig as an outgroup) exons are alternatively spliced and tend to have ω>1 in the mouse-rat comparison [30], and (v) the frequency of nonsynonymous SNPs in human genes is higher in alternative regions than in constitutive regions [37].

## Methods

### Definitions

In an alternatively spliced gene, constitutive regions are defined as the ones that are always exonic and coding, and alternative regions as the ones that are either coding or spliced out. An exon can be either completely constitutive, or completely alternative, or non-coding, or consist of constitutive, alternative and non-coding regions.

A local meta-alignment is a concatenate of all alignment fragments of a fixed type (for example, coding alternative regions) for one particular gene.

A global meta-alignment is the concatenate of local meta-alignments of a fixed type for all genes of a fixed group (for example, for all fast-evolving genes).

### Data

Human and mouse mRNA sequences were taken from the NCBI RefSeq database [38] and orthologs were identified and aligned as described previously [39].

Overall, 12356 pairs of orthologous human and mouse genes were considered. The data flow through the analysis pipeline is shown in Figure 1. Out of 12356 human genes, 5754 genes had more than one protein isoform in the EDAS database of alternatively spliced genes [40]. These proteins were mapped to the human-mouse mRNA alignments using Pro-Frame [41] and the results were parsed using a set of Perl scripts that identified constitutive and alternative coding fragments of the human genes and their reading frames. Alternatives confirmed by protein sequences and read in a single frame were identified in 3079 genes. We further restricted the dataset to 3029 pairs with more than 70% nucleotide identity between the human and mouse genes, as we doubted that the other ones were reliable.

2358 genes were selected for individual substitution rate analysis. These were the ones with both the total length of the human-mouse alignment length of the alternative regions and that of the constitutive regions exceeding 80 base pairs.

### Data classification

We grouped genes with comparable average substitution rates and formed three bins of equal size: slow, medium-speed, and fast-evolving genes.

We also considered alternative coding regions corresponding to protein N-terminal, middle, and C-terminal parts separately.

### Estimation of substitution rates

The transitional to transversional substitution rate ratio R, as well as thenumbers of synonymous (d_S_) and nonsynonymous (d_N_) substitutions per site were estimated by the Ina method I [32]. Unlike maximum likelihood methods, it afforded considerable results for very long alignments (~ 3·10^6 ^bp) and it proved to be fast enough to allow bootstrap resampling. We used our own implementation of this method (a set of Perl scripts).

### Bootstrapping

To evaluate the robustness of the estimates for evolutionary parameters of the global meta-alignments, we used bootstrapping to form 2000 alighments of the same length for reach global meta-alignment and estimated amino-acid identity, d_N_, d_S_, and ω = d_N_/d_S_.

## Authors' contributions

RN provided the EDAS database. EE and RN analysed the data. MG designed the project. EE and MG wrote the paper. All authors read and approved the final manuscript.

## Figures and Tables

**Table 1:** Substitution rates in constitutive and alternative regions of human-mouse concatenated alignments

Concatenate type	total alignment length, bp	amino-acid identity	d_N_	d_S_	ω
All genes					
C	2822439	0.891	0.068	0.409	0.166
A	3081642	0.879	0.077	0.410	0.187
AN	2194521	0.880	0.075	0.404	0.186
AI	790026	0.884	0.074	0.421	0.176
AC	97095	0.813	0.133	0.446	0.297
Slow-evolving genes					
C	897471	0.964	0.020	0.327	0.061
A	920970	0.960	0.023	0.324	0.071
AN	670623	0.961	0.022	0.323	0.068
AI	230754	0.957	0.024	0.327	0.075
AC	19593	0.940	0.036	0.329	0.111
Medium-speed evolving genes					
C	978984	0.913	0.052	0.422	0.123
A	1092459	0.903	0.059	0.420	0.139
AN	751620	0.903	0.059	0.413	0.142
AI	313896	0.905	0.057	0.434	0.131
AC	26943	0.887	0.073	0.455	0.160
Fast-evolving genes					
C	945984	0.800	0.135	0.480	0.281
A	1068213	0.785	0.147	0.481	0.306
AN	772278	0.788	0.143	0.474	0.302
AI	245376	0.787	0.148	0.504	0.294
AC	50559	0.724	0.209	0.493	0.425

C – constitutive regions

A – alternative regions

AN – N-terminal alternative regions

AI – internal alternative regions

AC – C-terminal alternative regions

R – the transition to transvertion rate ratio

d_N _– the nonsynonymous substitution rate

d_S _– the synonymous substitution rate

ω = d_N_/d_S_
